# Clinical Profiles and Mortality-Associated Risk Factors in Patients with Acute Kidney Injury from Atlixco, Puebla, Mexico

**DOI:** 10.3390/diagnostics15222889

**Published:** 2025-11-14

**Authors:** Nancy K. Zúñiga-Fernández, Pedro A. Gaspar-Mendoza, Lizeth Torres-Pineda, Elizabeth Baez-Baez, Karina Alvarado-Dardón, Karla V. Gutiérrez-de Anda, Jorge Ayón-Aguilar, Rubí Romo-Rodríguez, Rosana Pelayo, Diana Casique-Aguirre

**Affiliations:** 1 Emergency Department, Hospital General de Zona 5, Instituto Mexicano del Seguro Social, Atlixco 74360, Puebla, Mexico; nanzukafernandez@gmail.com (N.K.Z.-F.); karyad@live.com.mx (K.A.-D.);; 2 Laboratory of Oncoimmunology and Cytomics of Childhood Cancer, Centro de Investigación Biomédica de Oriente, Instituto Mexicano del Seguro Social, Atlixco 74360, Puebla, Mexico; pedro.a.gapar99@gmail.com (P.A.G.-M.); lizethtorrespineda553@gmail.com (L.T.-P.); elizabeth.baez@alumno.buap.mx (E.B.-B.); rubiromo2306@gmail.com (R.R.-R.); 3 Órgano de Operación Administrativa Desconcentrada (OOAD) Puebla, Instituto Mexicano del Seguro Social, Puebla 72089, Puebla, Mexico; jorge.ayona@imss.gob.mx; 4 Investigadoras e Investigadores por México, Secretaría de Ciencias, Humanidades, Tecnología e Innovación (SECIHTI), Benito Juárez, Ciudad de México 03940, Mexico; 5 Unidad de Educación e Investigación, Instituto Mexicano del Seguro Social, Alcaldía Cuauhtémoc, Ciudad de México 06725, Mexico

**Keywords:** acute kidney injury, imss, mortality risk, atlixco, COVID-19

## Abstract

**Background**: Acute Kidney Injury (AKI) is characterized by rising morbidity and mortality rates, along with significant financial costs associated with its treatment, positioning it as a priority health challenge. Difficult access to accurate biomarkers for renal dysfunction poses challenges in identifying high-risk patients prone to progression to severe AKI. Therefore, this study aimed to identify clinical and laboratory variables that could contribute to future risk stratification approaches in AKI. **Methods**: This observational retrospective study included 106 patients diagnosed with AKI who were admitted to the emergency department of the HGZ05-IMSS Hospital between January 2020 and July 2023. Multivariate logistic regression was used to identify clinical and laboratory factors associated with in-hospital mortality. **Results**: Patients with AKI exhibited elevated inflammatory indices (NLR, MLR, and PLR), increased levels of glucose, urea, and C-reactive protein (CRP), and reduced lymphocyte counts, serum albumin, FiO_2_, and BUN/creatinine (BCR) ratio. The hematological profile showed myeloid predominance, characterized by neutrophilia and lower eosinophil, erythrocyte, and monocyte counts, consistent with systemic inflammation. Multivariable analysis identified COVID-19 infection, thrombocytopenia, low eosinophil levels, and polypharmacy as independent predictors of mortality in AKI patients. **Conclusions**: These findings underscore the interplay between inflammatory, metabolic, and hematological alterations in AKI and highlight key prognostic factors that may contribute to future risk stratification.

## 1. Introduction

Acute kidney injury (AKI) is a sudden loss of kidney function that leads to the retention of nitrogenous waste products, such as creatinine [1]. It represents part of a continuum of kidney disorders that may result in the irreversible loss of renal cells and nephrons, potentially progressing to chronic kidney disease (CKD) [2].

Currently, the diagnosis of AKI is primarily based on the Kidney Disease: Improving Global Outcomes (KDIGO) [3] classification system, as well as the earlier Acute Kidney Injury Network (AKIN) [4] and Risk, Injury, Failure, Loss of Renal Function, and End-stage Renal Disease (RIFLE) [5] criteria. Although these definitions differ slightly, all rely on changes in serum creatinine and urine output, which have limited sensitivity for early injury. Serum creatinine rises only after a significant loss of renal function, often 24 to 48 h after injury onset, and can be affected by medications and other factors [6]. These limitations delay diagnosis and hinder timely intervention. Importantly, none of the current classification systems provide information regarding the specific etiology of the kidney injury [6,7].

To overcome these limitations, several emerging biomarkers, such as Cys-C, NGAL, L-FABP, KIM-1, IL-18, IGFBP-7, and TIMP-2, have been proposed to detect kidney injury earlier than conventional tests [8]. Although they hold promise for improving early diagnosis and risk stratification, they have not yet been incorporated into routine clinical practice [9].

These disorders have a significant impact on global health, and the World Health Organization (WHO) reporting a steady increase in kidney disease-related deaths from 813,000 in 2000 to 1.3 million in 2019. During this period, kidney disease rose from the 13th to the 10th leading cause of death worldwide. Prior to the COVID-19 pandemic, the mortality rate of patients with AKI in Mexico was approximately 25% [10,11]. By 2021, kidney disease had become the sixth leading cause of death in the country [12]. However, the lack of a national disease registry indicates that the actual burden may be even greater, particularly in critically ill patients [11]. In the state of Puebla, for instance, there are no specific reports on AKI; nevertheless, available data on CKD provide an indirect approximation to the regional situation, since both conditions share similar risk factors and environmental determinants. CKD mortality rates in Puebla exceed the national averages, with younger populations being particularly vulnerable. For example, between 2019 and 2021, CKD-related deaths among individuals aged 10–19 years were 0.8 per 100,000, compared with the national rate of 0.7 per 100,000, representing a mortality risk 1.3-fold higher than the national average [13].

AKI is a critical prognostic factor in hospitalized patients, particularly those in intensive care units (ICUs), where its incidence is on the rise [2,14,15]. In developing countries, AKI contributes to approximately 15% of hospital admissions, with Mexican tertiary care centers reporting that 64% of ICU patients develop AKI [11]. This condition complicates the management of chronic diseases, reduces survival rates, and is strongly associated with worse outcomes, including prolonged hospital stays and higher mortality [16,17,18]. COVID-19 pandemic has further highlighted these challenges, as AKI has emerged as a significant risk factor for poor prognosis [19,20].

These potential correlations, coupled with the limited availability of biomarkers for AKI diagnosis, highlight the need for early evaluation to detect kidney damage in this population group. This study aimed to analyze the clinical profile of AKI in a mixed cohort that reflected the diversity of cases observed during the pandemic period at the Hospital General de Zona 5 (HGZ05), IMSS, Atlixco, Puebla, Mexico. AKI classification followed according the KDIGO guidelines. Clinical data were statistically analyzed to determine the risk profiles of these populations.

## 2. Materials and Methods

This study was approved by the local IMSS Health Research and Ethics Committee (Registry R-2021-2106-019) and adhered to the national and international ethical guidelines for research involving human participants.

Study design and participants. This retrospective observational study analyzed the clinical records of 106 patients diagnosed with AKI admitted to the Hospital General de Zona No. 5 (HGZ05) between January 2020 and July 2023. Eligible patients were between 18 and 90 years of age, of either sex (55 men and 51 women). Patients with incomplete medical records, insufficient data for AKI classification, or a previous diagnosis of CKD were excluded from the analysis.

AKI classification. Patients were selected based on the presence of AKI. For each patient, at least three laboratory reports showing alterations in renal function during hospitalization were reviewed. Admission serum creatinine values were considered as baseline for KDIGO classification. Patients were classified according to the 2012 KDIGO [3], which define AKI severity in three stages (I–III) based on serum creatinine levels and urine output. Stage I: Creatinine increase ≥ 0.3 mg/dL or 1.5–1.9-fold baseline; urine output < 0.5 mL/kg/h for 6–12 h. Stage II: Creatinine 2–2.9-fold baseline; urine output < 0.5 mL/kg/h for <12 h. Stage III: Creatinine ≥ 3-fold baseline or ≥4 mg/dL, urine output < 0.3 mL/kg/h for 24 h or anuria for 12 h.

Data collection. All demographic and clinical data were obtained from the patients’ clinical records. The variables analyzed included: age at diagnosis, sex, comorbidities (diabetes mellitus, hypertension, COVID-19, and recurrent urinary tract infections (rUTI)), polypharmacy (use of ≥3 drugs), and AKI classification (stage I, stage II, or stage III).

The clinical parameters included serum creatinine levels at admission and subsequent measurements during hospitalization, with at least 24 h intervals. Laboratory tests included complete blood count (glucose, urea, blood urea nitrogen [BUN], blood urea nitrogen-to-creatinine ratio [BCR], C-reactive protein [CRP], fibrinogen, serum calcium, serum albumin, serum chloride, pH, partial pressure of carbon dioxide [pCO_2_], partial pressure of oxygen [pO_2_], bicarbonate [HCO_3_^−^], oxygen saturation (%), and fractional inspired oxygen [FiO_2_]).

Inflammatory markers were obtained using the data recorded in the patients’ complete blood count records, such as the neutrophil-to-lymphocyte ratio (NLR), which resulted from dividing the neutrophil count by the lymphocyte count; the platelet-to-lymphocyte ratio (PLR), from dividing the platelet count by the lymphocyte count; and the monocyte-to-lymphocyte ratio (MLR), from dividing the monocyte count by the lymphocyte count [21,22,23].

Patients were grouped according to age (young: 18–30 years, adults: 31–60 years, elderly: 61–90 years old) and discharge outcomes (resolution, death, voluntary discharge).

Statistical analysis. Continuous variables were compared across AKI stages using one-way ANOVA.

Univariate Analysis. To assess the association between variables and mortality, an odds ratio (OR) analysis was performed, considering four groups: (1) patients who died and presented the variable, (2) patients who died and did not present the variable, (3) patients who experienced AKI resolution and presented the variable, and (4) patients who experienced AKI resolution and did not present the variable. Odds ratios and 95% confidence intervals (CI) were calculated from 2 × 2 contingency tables using the Woolf logit method. All statistical analyses were performed using GraphPad Prism 6 (La Jolla, CA, USA). Patients who were discharged voluntarily were excluded from the analysis because their outcomes were uncertain.

Multivariate Analysis. Hospital mortality (1 = death, 0 = survival) was modeled using logistic regression with L1 (lasso) penalization to limit overfitting given the events-per-variable ratio. The model was implemented using the fit_regularized function. The regularization parameter (α = 0.01) was selected after evaluating model convergence and coefficient stability across a range of penalization values (0.001–0.1). Candidate variables were selected through univariable screening (*p* < 0.05). Variables with excessive missing data were excluded to maintain model stability, while clinically relevant factors, COVID-19, hypertension, and diabetes were forced into the model as potential confounders, regardless of their *p*-value in the univariable analysis. Missing categorical values were imputed using the mode of each variable, an appropriate approach for binary predictors with limited missing data (<5%). Adjusted odds ratios (aOR) with 95% confidence intervals (95% CI) were derived from the penalized model. Model discrimination was assessed using the area under the receiver operating characteristic (ROC) curve (AUC) and 5-fold cross-validation (mean ± SD), and calibration was evaluated using the Brier score. For binary classification, the operational threshold was defined by Youden’s index, reporting sensitivity, specificity, PPV, and NPV at that threshold. Analyses were performed in Jupyter-Python (version 6.4.12) (statsmodels, scikit-learn).

To visualize the categorical and qualitative parameters for each AKI stage, alluvial diagrams were generated using RStudio (version 2024.04.2+764) with the ggalluvial and ggplot2 libraries (version 3.5.1). Bar graphs with mean values and standard deviations (SD) were used to summarize the numerical data. Comparisons between groups were performed using an unpaired *t*-test with Welch’s correction. Statistical significance was set at *p* < 0.05.

## 3. Results

### 3.1. Clinical Profiles in Patients with Acute Kidney Injury

**Figure A1:**
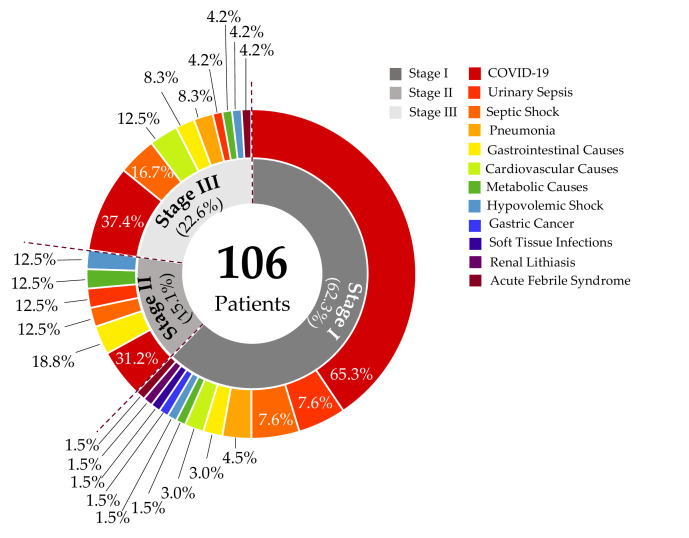
Admission diagnoses for patients with Acute Kidney Injury in emergency care. The most prevalent diagnoses in patients who develop AKI are categorized as stages I, II, and III. These include various conditions such as COVID-19, urinary sepsis, septic shock, pneumonia, gastrointestinal causes, metabolic causes, cardiovascular causes, hypovolemic shock, gastric cancer, soft tissue infections, renal lithiasis, and acute febrile syndrome.

This study included 106 patients who were admitted to the emergency department of HGZ05. Among them, 62.3% were classified as stage I, 15.1% as stage II, and 22.6% as stage III patients. COVID-19 was the most common diagnosis at admission, followed by septic shock, urinary sepsis, and gastrointestinal disorders (Figure A1).

A correlation was observed between advanced age and AKI severity. Stage I patients had the highest recovery rate (74.2%), whereas those with stage III disease had the highest mortality rate (54.2%). Of the cohort, 53.8% were diagnosed with COVID-19, and 48.1% were on polypharmacy regimens. Hypertension (44.3%) and diabetes (42.5%) were the most common comorbidities, particularly in stage-III patients. Overweight status (14.2%) and recurrent urinary tract infections (rUTI) (13.2%) were more frequently observed in stage II (Table 1).

At admission, the patients exhibited an average serum creatinine level of 1.86 mg/dL, which decreased to 1.44 mg/dL during their stay. However, patients with stage II and III diseases did not regain normal creatinine levels. Leukocyte counts remained near the upper normal limit, with elevated neutrophil counts and consistent lymphopenia across all stages (Table 1).

Inflammatory markers such as the NLR, MLR, and PLR, were elevated, with values 4.2-, 1.3-, and 1.8-fold higher than normal, respectively. Blood glucose levels were high, particularly in patients with stage III disease, who exhibited levels that were 2.1-fold higher than the reference value. Concentrations of blood urea and blood urea nitrogen also surpassed normal values, with stage III patients displaying the most significant elevations, at 2.5-fold the normal limit for both parameters. The CRP level, an inflammatory marker, exceeded the normal range in all stages, averaging 6.9-fold higher than normal values. In addition, most patients presented hypoalbuminemia. The FiO_2_ required increased, reaching 2.7-fold the normal value in stage I patients (Table 1).

### 3.2. Factors Associated with Mortality

Patients discharged voluntarily were excluded because their outcomes were uncertain. Of the 106 patients initially enrolled, 97 were included in the final analysis (35 deaths and 62 survivors).

**Figure A2:**
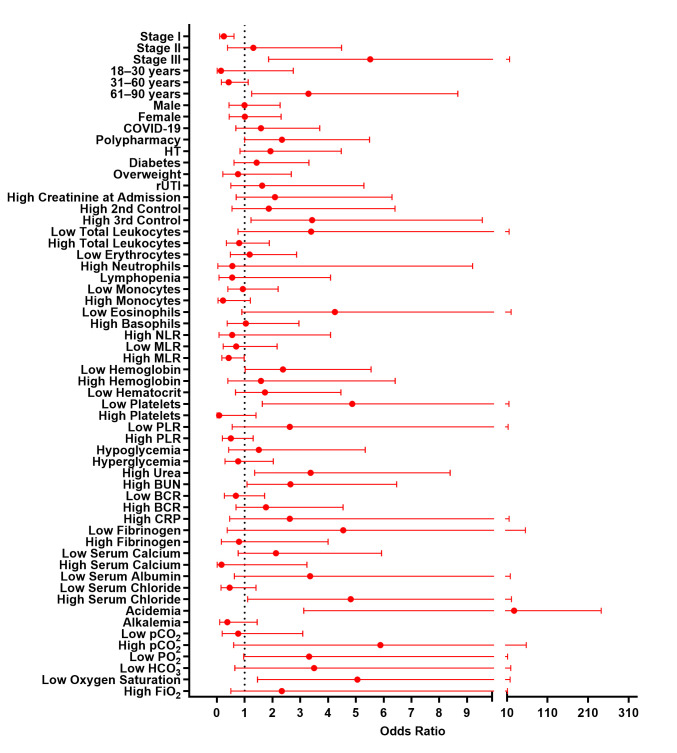
Univariable analysis of factors associated with in-hospital mortality in AKI.

**Figure A3:**
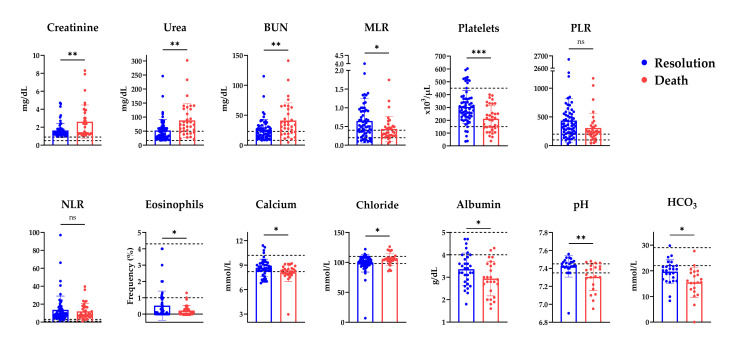
Disease resolution and death outcomes according to the quantitative parameters. Comparison of laboratory parameters among patients with AKI with outcomes of disease resolution or death. Reference values: creatinine 0.5–1.1 mg/dL, urea 16.6–49.5 mg/dL, BUN 8–23 mg/dL, MLR 0.23, platelets 150–450 × 10^3^/μL, eosinophils 1–4.3%, calcium 8.2–10.2 mmol/L, chloride 98–110 mmol/L. BUN: Blood Ureic Nitrogen;MLR, monocyte-to-lymphocyte ratio; PLR: Platelet-to-lymphocyte ratio;NLR: Neutrophil-to-lymphocyte ratio; * *p* ≤ 0.05; ** *p* ≤ 0.01; *** *p* ≤ 0.001; ns: not significant.

Univariable analysis revealed variables significantly associated with mortality, including stage III AKI, which increased the risk by 5.5-fold. All patients aged ≤30 years recovered successfully, whereas most deaths occurred in patients aged >60 years (Table 1). Advanced age (61–90 years) was associated with an increased risk (OR = 3.29; 95% CI, 1.25–8.67; *p* = 0.0158), reflecting the predictable vulnerability of this population. Thrombocytopenia (low platelet count) (OR = 4.87; 95% CI, 1.63–14.54; *p* = 0.0054), elevated urea (OR = 3.37; 95% CI, 1.35–8.39; *p* = 0.0099), and elevated BUN (OR = 2.64; 95% CI, 1.08–6.42; *p =* 0.0338) were also associated with an increased risk of mortality. Acidemia (OR = 27.5; 95% CI, 3.12–242.1; *p* = 0.0002) and low oxygen saturation (OR = 5.05; 95% CI, 1.45–17.53; *p* = 0.0173) emerged as additional contributors (Figure A2 and Table A1). Additionally, non-survivors exhibited lower MLR and HCO_3_^−^ levels compared to survivors (Figure A3).

In the multivariable analysis, COVID-19 (aOR 3.68; 95% CI, 1.10–12.29; *p* = 0.0343), low platelets (aOR 7.06; 95% CI, 1.71–29.18; *p* = 0.0069), low eosinophils (aOR 22.46; 95% CI, 2.18–231.61; *p* = 0.0089), Stage III (aOR 8.24; 95% CI, 1.76–38.57; *p* = 0.0074), and polypharmacy (aOR 3.86; 95% CI, 1.15–12.97; *p* = 0.0289) were identified as independent factors significantly associated with AKI-related mortality.

Other variables such as high urea, hypertension, diabetes, and age > 60 did not retain significant association after adjustment.

**Figure 1:**
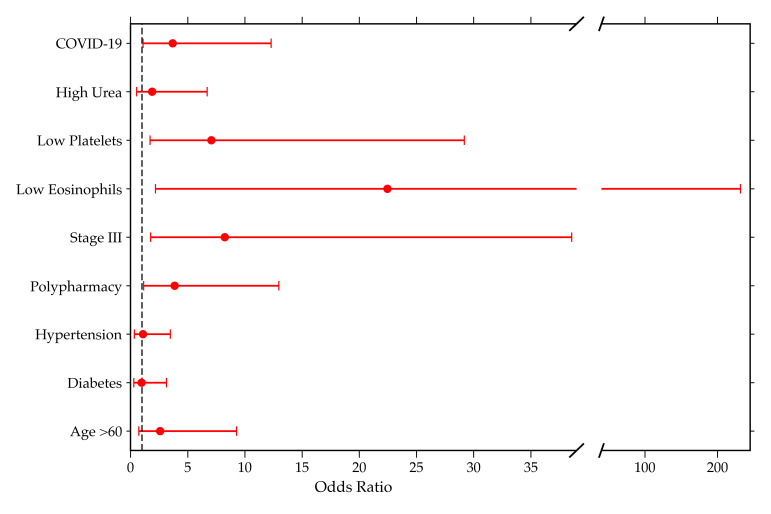
Forest plot showing adjusted odds ratios (aOR) and 95% confidence intervals of variables associated with AKI-related mortality.

Table 2 and Figure 1 show the aOR and 95% confidence intervals on a logarithmic scale (forest plot), highlighting the main factors associated with in-hospital mortality among patients with AKI.

**Figure A4:**
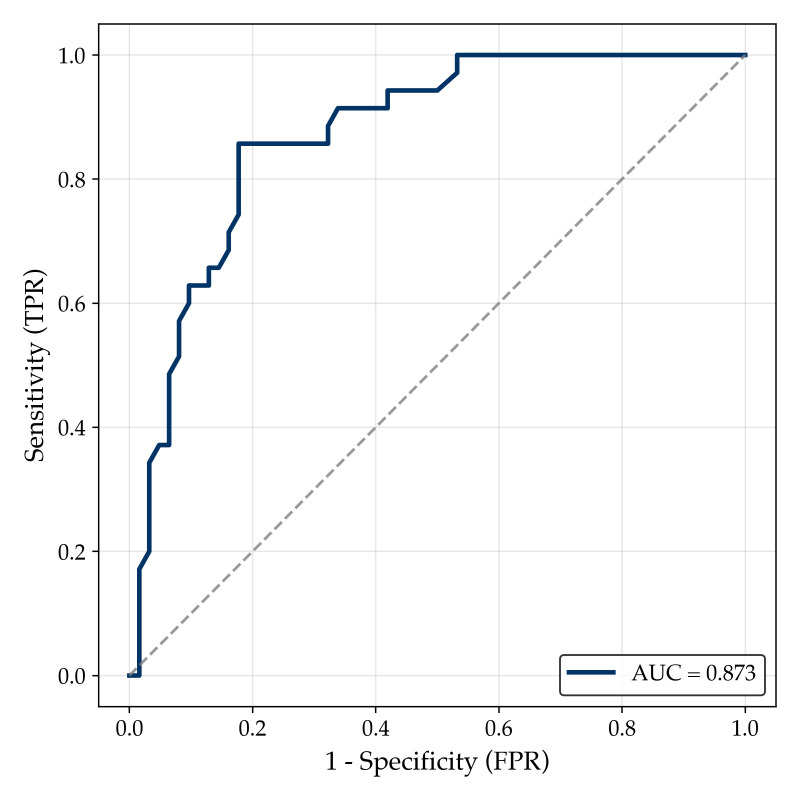
Receiver operating characteristic (ROC) curve of the multivariable logistic regression model for mortality prediction in AKI patients. The ROC curve illustrates the discriminative ability of the multivariable logistic regression model to predict in-hospital mortality in patients with AKI. The model achieved an AUC of 0.873, indicating excellent performance. The dashed diagonal line represents the line of no discrimination (AUC = 0.5).

The multivariable logistic regression model demonstrated excellent discriminative ability, with an area under the receiver operating characteristic (ROC) curve (AUC) of 0.873 (Figure A4). Bootstrap validation confirmed the model’s performance, showing an AUC of 0.872 (95% CI, 0.798–0.936), and 5-fold cross-validation yielded an average AUC of 0.796 ± 0.145, indicating adequate model stability. Calibration, evaluated by the Brier score (0.139), reflected good agreement between predicted probabilities and observed outcomes.

The optimal threshold defined by Youden’s index (0.403) achieved a sensitivity of 0.857, specificity of 0.823, positive predictive value (PPV) of 0.732, and negative predictive value (NPV) of 0.911, supporting the model’s usefulness in identifying patients at high risk of mortality (Table A2).

#### Quantitative and Categorical Parameters Suggest Death Risk Profiles for AKI Stages II and III

**Figure 2:**
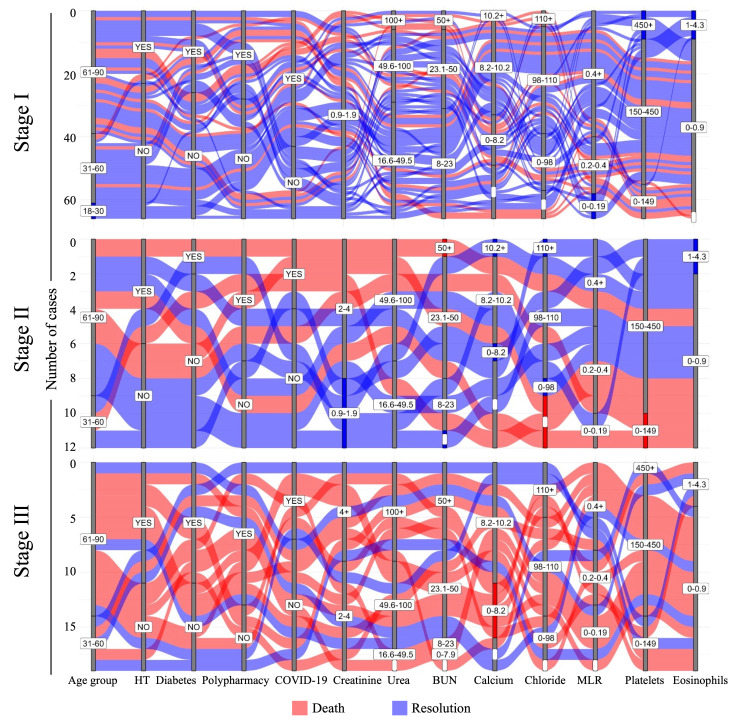
Fate mapping of AKI stages to predict death-risk profiles. Alluvial plots for laboratory and clinical parameters in patients with AKI were categorized according to the stage of AKI. Each case is represented by a single strip in the figure. Patients with disease resolution are represented in blue, and fatal cases are represented in red. MLR: monocyte-to-lymphocyte ratio; HT: hypertension; White bars indicate missing data.

To depict a potential risk profile, alluvial diagrams were created using clinical and laboratory data from patients with AKI (*n* = 97) whose disease resolution was known, enabling the simultaneous visualization of multiple parameters according to the disease stage of each case. Patients within the cohort who resolved the acute disease in the short term are represented in blue, whereas cases with fatal outcomes are shown in red. Quantitative parameters were stratified based on reference values, and categorical parameters were stratified according to the presence or absence of comorbidities (Figure 2).

Nitrogenous products, such as urea and BUN, were within the reference limits in more than half of the patients in stage I, in contrast to the few cases in stage III. The dynamics of the cellular populations also marked the transition between stages of the disease from good to poor prognosis. A substantial proportion of stage III patients exhibited MLR values outside the normal range, and nearly 70% of individuals with a favorable prognosis maintained their platelet counts within the reference range. Approximately one-third of patients with severe disease showed very low platelet counts. Patients with platelet counts exceeding the reference values survived, regardless of whether they were diagnosed with stage I or III disease (blue strata). In contrast, normal eosinophil and elevated calcium levels were associated with survival (Figure 2).

From an integrative perspective of AKI classification groups in relation to mortality, it was observed that most patients across the three stages were >60 years old and showed a trend toward certain clinical characteristics. These include elevated concentrations of urea and BUN, reduced eosinophil counts, and comorbidities such as COVID-19, diabetes, hypertension, and polypharmacy. A portion of the population that died exhibited decreased platelet counts, which was significant in patients with stage II disease. Additionally, elevated chloride levels were found in stage I, stage II was characterized by a decrease in platelet counts, and stage III by a low concentration of calcium (Figure 2).

## 4. Discussion

The epidemiological landscape of AKI in Mexico remains a health challenge, primarily due to the lack of a national disease registry and standardized definitions guiding the accurate assessment of AKI severity. Current data are often reported using varying classifications systems, such as kidney disease: KDIGO, AKIN, and RIFLE. Additionally, the absence of precise and widely accessible biomarkers further limits the ability to achieve early diagnosis and reliable prognosis of AKI [11]. Therefore, identifying clinical determinants involved in the onset of AKI is of great importance for improving early detection and timely management.

**Figure 3:**
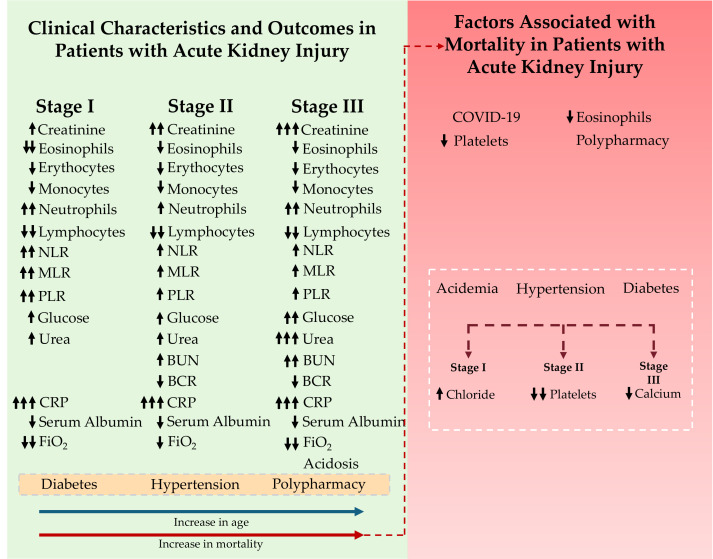
Overview of the study population and factors associated with mortality in patients with acute kidney injury (AKI). The green section presents the clinical features and outcomes across different AKI stages, showing progressive increases in creatinine, inflammatory markers, glucose, urea, and CRP, along with reductions in lymphocytes, serum albumin, and FiO_2_. Advanced stages (II and III) exhibit further elevations in urea and BUN levels, reflecting the progression of the disease. Comorbidities such as diabetes and hypertension, as well as polypharmacy, are frequently observed among patients with AKI. As age increases, the likelihood of developing more advanced AKI stages (blue arrow) and the probability of death (red arrow) also rise. The right section presents the mortality-associated factors and their categorization according to the AKI stage. The white dotted region indicates the mortality trend. Black arrows next to each variable indicate its direction of change across AKI stages or the direction of its association with mortality (↑ increase/higher odds; ↓ decrease/lower odds).

This study analyzed a cohort of 106 patients diagnosed with AKI who were admitted to the emergency department of the Hospital General de Zona No. 5 (IMSS), aiming to characterize their clinical profiles and identify key determinants associated with patient mortality (Figure 3).

Currently, Atlixco, Puebla has a population of 141,793 inhabitants, according to the most recent official census. The average level of education is 9.4 years, and approximately 3568 individuals speak Indigenous languages [28]. The local healthcare infrastructure includes one primary care IMSS clinic and two public secondary-level hospitals: the General Hospital of Atlixco Gonzalo Río Arronte and the General Hospital of Zone No. 5-IMSS, which have 45 and 43 available beds, respectively. The region lacks tertiary-level hospital services. Given these characteristics, the population included in this study can be considered representative of the Atlixco region. In this context, identifying accessible and clinically useful features to anticipate the development of AKI is a priority particularly for populations with limited access to advanced diagnostic tools.

### 4.1. Clinical Profile of Patients with AKI

Analysis revealed a positive correlation between advanced age and disease severity. No cases of stage III AKI or associated fatalities were observed in patients aged <31 years. The increased susceptibility of older adults to AKI may be explained by age-related anatomical and physiological changes in kidney function, including reduced renal blood flow and decreased glomerular filtration rate. Of note, at this age, disease progression appears to relate to multiple comorbidities and polypharmacy [29].

Continuous monitoring of serum creatinine levels is crucial for detection and management of AKI. This process, known as “creatinine follow-up,” typically involves measurements taken at intervals of 24 h or less. Patients with stage I AKI demonstrate renal function recovery. However, in stages II and III, recovery was either prolonged or incomplete. Poor recovery in the advanced stages is likely due to ischemia, acute tubular necrosis, and free radicals, which are further exacerbated by additional risk factors [30,31,32]. Moreover, patients exhibited low eosinophil, erythrocyte, and monocyte counts, along with elevated neutrophil counts. Taken together, these findings indicate a myeloid lineage imbalance consistent with systemic inflammation and increased disease severity in patients with AKI.

PLR, MLR, and NLR are established indicators of inflammation and predictors of AKI in ICU patients. Although previous studies have shown an association with mortality [33,34,35], we did not identify a significant relationship between these parameters and mortality. However, neutrophil counts, PLR, MLR, and NLR were elevated above the reference values in this cohort, suggesting their potential use as indicators of AKI. This has been widely reported in the Chinese population [35,36].

Two retrospective studies have evaluated the prognostic value of inflammation-based biomarkers in patients with AKI in intensive care settings. In a study conducted at the Second University Hospital of Lanzhou between January 2015 and January 2017, 120 patients diagnosed with sepsis and AKI (defined according to KDIGO criteria) were included. The analysis revealed a significant association between the PLR and mortality (OR = 1.021; 95% CI: 1.003–1.039; *p* < 0.05), suggesting that elevated PLR may be linked to a poorer prognosis in sepsis-induced AKI [35]. Similarly, a larger study at the First Affiliated Hospital of the University of South China retrospectively analyzed clinical records of 1500 ICU patients admitted between January 2016 and December 2019. A total of 615 patients (41%) developed AKI based on KDIGO criteria. Both the MLR and NLR were positively correlated with AKI incidence (*p* < 0.001). These markers showed predictive performance for AKI, with areas under the curve (AUC) of 0.899 for MLR and 0.780 for NLR. However, their utility for predicting in-hospital mortality was limited, with AUCs of 0.583 for MLR and 0.564 for NLR, indicating poor discriminative capacity for this outcome [34]. Similarly, our findings suggest that while MLR and NLR may be helpful in identifying patients at risk for AKI, their effectiveness as independent predictors of mortality in critically ill patients is limited.

Among hospitalized patients, pre-existing conditions such as hypertension, diabetes, and COVID-19 have been widely recognized as key risk factors for the development of AKI [37,38,39,40]. Within Latin America, a multicenter registry including 870 hospitalized patients with COVID-19–related AKI across 12 countries reported an in-hospital mortality of 62.5%, renal replacement therapy in 46.2%, and lack of renal recovery in 65.3% of cases. These adverse outcomes were more frequent than in our cohort, likely reflecting differences in disease etiology (mixed AKI vs. COVID-19–only populations), baseline severity, and healthcare resource availability. Nevertheless, this study underscores the substantial burden of AKI in Latin America and highlights the importance of generating regional data to improve risk stratification and management strategies [41]. Hyperglycemia, a well-known risk factor, causes endothelial damage and induces renal microvascular alterations impairing kidney function. These effects are not limited to diabetic individuals, as non-diabetic patients with uncontrolled blood glucose levels may also be affected [42]. This study found that 75.5% of patients presented with hyperglycemia, while only 41.6% had a prior diagnosis of diabetes, highlighting the potential role of hyperglycemia as a contributor to renal injury. This underscores the importance of close glycemic monitoring in all individuals to reduce the risk of AKI.

Of interest, sequential increase in blood urea and BUN levels was observed as the disease progressed, as well as elevated levels of creatinine, urea, and BUN, which is consistent with the pathophysiology of AKI [43,44]. Renal replacement therapy (RRT) may play a crucial role in this context. Its implementation in the presence of elevated BUN levels could help reduce these values and potentially mitigate clinical complications in patients with AKI [44,45]. Accordingly, BCR reduction (<20) was observed in patients with stage II and III AKI. Supporting this association, a study conducted at the General Hospital of Zacatecas, Mexico, included 42 patients with a mean age of 45 years, all diagnosed with AKI according to KDIGO criteria. Among them, 19 patients exhibited histological findings consistent with acute tubulointerstitial nephritis (ATIN). A significant inverse correlation was identified between BCR and the presence of ATIN (r = −0.57; *p* = 0.001). ROC curve analysis determined an optimal BCR cutoff value of ≤12 for classifying ATIN, with an AUC of 0.73 (*p* = 0.024). According to the authors, this cutoff yielded a sensitivity of 76%, specificity of 81%, and an OR of 14 (95% CI: 2.6–75.7; *p* = 0.021) [46]. These findings suggest that the BCR may serve as a clinical marker with prognostic value in patients with AKI, given its significant correlation with histologically confirmed renal damage.

Polypharmacy is common among patients with comorbidities such as diabetes and hypertension. In this study, 48.1% of patients with AKI reported chronic use of multiple medications. Chronic intake of multiple drugs places additional stress on the kidneys, requiring them to work harder to excrete a wide range of drugs and their metabolites, potentially leading to burden and impaired renal function [47]. Supporting this, a study conducted at the National Taiwan University Hospital (NTUH) investigated the association between cardiovascular (CV) polypharmacy and the risk of AKI in 152 patients over the age of 60 who were admitted to general medicine wards. Based on KDIGO classification, 48% of patients developed AKI, and 64% had been exposed to CV polypharmacy. The incidence of AKI increased progressively with the number of cardiovascular medications taken prior to admission (0 medications: 33%; 1 medication: 50%; 2 medications: 57%; ≥3 medications: 60%; *p* = 0.05). Furthermore, patients with more severe stages of AKI were more likely to have been taking multiple cardiovascular drugs before hospitalization. The analysis revealed that the use of cardiovascular medications was associated with a greater risk of AKI upon admission (1 medication: OR = 1.63, *p* = 0.2; 2 medications: OR = 4.74, *p* = 0.03; ≥3 medications: OR = 5.92, *p* = 0.02). Cardiovascular polypharmacy, when treated as a categorical variable, also showed a significant association with AKI risk (OR = 2.58; *p* = 0.02), and each additional cardiovascular medication was found to increase the risk of AKI by approximately 30% [48]. These findings highlight the importance of moderate and well-monitored use of medications to help reduce the risk of renal injury.

According to 2023 data obtained from the Comité Estatal de Información Estadística y Geográfica del Estado de Puebla (CEIGEP, https://ceigep.puebla.gob.mx/fichas/descargas/19/ATLIXCO, accessed on 11 November 2025), in Atlixco the leading chronic causes of death include diabetes mellitus (232 deaths) and acute myocardial infarction (214 deaths), followed by chronic liver disease (91 deaths) and chronic kidney failure (49 deaths). These conditions commonly require long-term multidrug therapy, which increases the likelihood of polypharmacy. Notably, 37.6% experiences limited access to medical care, condition that may exacerbate the risks associated with chronic disease management and self-medication. Considering this clinical and social profile, the estimated prevalence of polypharmacy among patients with chronic diseases in Atlixco is plausibly high, underscoring the need for rational medication use and strengthened pharmacovigilance in this region.

### 4.2. Predictors of Mortality Among Patients with AKI 

This study identified several factors potentially related to mortality in patients with AKI, COVID-19, low platelets, low eosinophils, stage III, and polypharmacy. Furthermore, in this cohort, a higher prevalence of acidemia was observed in patients who progressed to stage III AKI, highlighting its possible role in disease severity and outcome.

A retrospective study conducted in adult patients with COVID-19, hospitalized within the Mount Sinai Health System, the largest hospital network in New York State, between 27 February and 30 May 2020, evaluated the frequency and severity of AKI using the KDIGO criteria. Out of a total of 3993 hospitalized patients, 1835 (46%) occurred AKI. Among these, the proportions corresponding to AKI Stages I, II, and III were 39%, 19%, and 42%, respectively. In-hospital mortality was higher among patients with AKI (50%) compared to those without AKI (8%). Additionally, only 36% of patients with AKI survived and recovered renal function by the time of discharge [49]. These findings reinforce the role of COVID-19 as a major contributor to AKI development during hospitalization and highlight its impact on in-hospital mortality—trends that align with those observed in our study.

The association between thrombocytopenia and mortality in patients with AKI has been previously reported [50]. In a retrospective study conducted at the Civil Hospital of Guadalajara, Jalisco, Mexico, between 2017 and 2023, a total of 400 patients diagnosed with AKI according to KDIGO criteria were included. Among them, 134 patients exhibited a platelet count reduction greater than 21%, with a mean age of 54 years. The risk of mortality was found to be three times higher in patients with a significant decrease in platelet count (<90 × 10^3^ cells/µL) (OR: 2.9; 95% CI: 1.1–7.7; *p* = 0.02), suggesting a strong association between thrombocytopenia and poor clinical outcomes in patients with AKI [51].

Although few studies have focused exclusively on eosinopenia in patients with AKI, evidence from ICUs and from patients with severe kidney injury indicates that this condition is associated with a worse prognosis. For example, a study conducted at Seoul National University analyzed 2823 patients admitted to the ICU of Bundang Hospital between June 2004 and June 2010 and reported that eosinopenia was an independent predictor of mortality among critically ill patients [52].

Regarding the trend of variables associated with mortality, a retrospective study conducted at the Seoul National University Hospital, a total of 2397 patients who underwent continuous renal replacement therapy (CRRT) for AKI between June 2010 and December 2020 were included. Patients were classified into five groups: two groups with stable pH (first group: pH = 7.4; second group: pH = 7.3), a recovery group (third group: pH from 7.2 to 7.3), a deterioration group (fourth group: pH from 7.3 to 7.2), and a group with persistent severe acidemia (fifth group: pH < 7.2). During a 10-day follow-up period, 1193 patients (65.7%) died. Mortality rates increased progressively across the five groups, with values of 44.3%, 55.7%, 74.2%, 78.2%, and 82.2%, respectively (*p* < 0.001). These findings suggest that worsening acidemia is associated with an increased risk of mortality in patients with AKI [53].

According to the results of this study, the variables associated with mortality differed by AKI stage. In stage I, elevated chloride levels showed a trend toward higher mortality. In stage II, thrombocytopenia emerged as a critical determinant, whereas in stage III, low serum calcium concentrations were significantly associated with increased mortality (Figure 3). Supporting the clinical relevance of electrolyte imbalances, a retrospective study of 250 adult patients admitted to the ICU of King Edward VIII Hospital, located in Durban, KwaZulu-Natal Province, South Africa, between 26 September 2015, and 9 May 2016, hyperchloremia was observed in 143 patients (57.2%) within the first 48 h of ICU admission. Hyperchloremia during this period was significantly associated with the development of AKI (OR = 6.44; 95% CI: 2.95–14.10) as well as with an increased risk of mortality (OR = 2.46; 95% CI: 1.22–4.94). AKI was diagnosed based on KDIGO criteria [54]. Similarly, a retrospective study conducted at the Mayo Clinic in Rochester, Minnesota, USA, which included all hospitalized adult patients from 1 January 2009 to 31 December 2013, reported that hypocalcemia was common in patients with AKI, particularly in KDIGO stages II and III [55]. Furthermore, a multicenter study across 27 critical care centers in the United States, which included adult patients (≥18 years) with AKI, identified severe hypocalcemia as an independent predictor of mortality in patients requiring renal replacement therapy [56].

The clinical variables and factors associated with the development and severity of AKI in this study were consistent with previous reports from other populations, reinforcing the robustness and generalizability of our findings despite regional limitations and underlying socio-environmental vulnerabilities.

### 4.3. Pathophysiological Mechanisms Driving the Development of Acute Kidney Injury (AKI)

The presence of COVID-19 in patients has emerged as a key determinant associated with the development and mortality of AKI, with its pathophysiology involving multiple mechanisms. One of the main mechanisms involved is systemic inflammation, in which cytokines such as IL-6 and TNF-α trigger an immune response that damages various tissues, including the kidneys [57,58]. Another proposed mechanism is direct viral injury to renal cells, as SARS-CoV-2 uses the ACE2 receptor, which is highly expressed in proximal tubular epithelial cells and podocytes. This interaction also disrupts the renin–angiotensin–aldosterone system, facilitating viral entry and replication in multiple tissues, including the renal parenchyma. Glomerulonephritis, renal hypoperfusion, hypoxia, and rhabdomyolysis have all been associated with kidney damage in COVID-19 patients. Additionally, some drugs used in COVID-19 treatment have nephrotoxic potential and may exacerbate renal injury. Furthermore, the virus-induced hypercoagulable state promotes the formation of microthrombi in glomerular capillaries, in association with endothelial dysfunction and complement activation [58,59].

The clinical features and progression across the different stages of AKI show a progressive increase in serum creatinine and BUN levels, due to the rapid decline in glomerular filtration rate (GFR), resulting in the accumulation of nitrogenous waste products [60]. Advanced stages (II and III) exhibit higher elevations in urea and BUN, reflecting disease progression.

Although literature specifically addressing eosinopenia in AKI is limited, observations in critically ill populations suggest that eosinopenia predominantly reflects an acute stress-response rather than chronic immunosuppression. Both endogenous and therapeutic corticosteroids can transiently reduce circulating eosinophils through CXCR4-dependent bone-marrow homing, indicating systemic stress and inflammatory burden rather than persistent immune dysfunction [61,62,63].

In 2020, the first study was published demonstrating a direct association between AKI and the development of long-term anemia. This relationship may be explained by persistent interstitial damage and reduced erythropoietin (EPO) production [64]. However, further research is needed to better understand this connection.

The observed reduction in monocytes may be explained by their active migration to the kidney tissue damaged by AKI, where they differentiate into macrophages. Recruited macrophages, initially of the pro-inflammatory Ly6C^hi^ (M1) subtype, are known to exacerbate tissue injury [65]. However, within the first five days after injury, these macrophages progressively lose Ly6C expression and adopt a reparative (M2) phenotype, after which their numbers decline [65,66]. Ly6C^low^ M2 macrophages are considered beneficial for renal tissue repair. In addition to clearing cellular debris and apoptotic neutrophils, macrophages also play a role in nephrogenesis [67,68]. During the recovery phase, M2 macrophages secrete factors such as Wnt-7b, fibronectin, growth factors, and IL-1 receptor antagonists, which promote epithelial regeneration [68,69]. Studies in murine models have shown that depleting macrophages prior to injury may be protective; however, their depletion during established AKI impairs M2-mediated repair and prolongs tissue damage [67,70]. This highlights the essential role of macrophages in renal recovery.

Moreover, in each AKI stage, an increase in various inflammatory markers (NLR, MLR, and PLR) was recorded, due to microvascular endothelial injury and alterations in the glycocalyx, which lead to endothelial cell activation and the expression of surface markers that promote leukocyte and platelet recruitment and adhesion [67,71]. Neutrophils infiltrate post-ischemic kidneys, where they primarily exert pro-inflammatory functions and contribute to kidney injury through the production of reactive oxygen species and cytokines, including IL-17 and IFN-γ [72,73].

In patients with AKI, levels of the active form of vitamin D, 1,25-dihydroxyvitamin D, are reduced. This deficiency contributes to impaired calcium absorption in the intestine, decreased renal calcium reabsorption, and a subsequent decline in serum calcium levels [74].

### 4.4. Limitations and Future Directions

1. The retrospective design of this study inherently limits the analysis due to potential biases, particularly those related to incomplete or missing data.
2. The relatively small sample size (*n* = 106) may limit the statistical power and generalizability of the findings. However, the inclusion of a multivariable logistic regression model with excellent discriminative and calibration performance (AUC = 0.873; Brier score = 0.139) supports the internal validity and reliability of the results. Larger and more diverse cohorts are still needed to confirm these findings and to extend their applicability to broader populations.
3. These findings should be interpreted considering that 53.8% of the patients were admitted with a diagnosis of COVID-19, while the remaining cases were associated with other causes of AKI, such as sepsis and gastrointestinal disorders. Therefore, the results reflect a mixed population of COVID-19 and non-COVID-19 patients.
4. Another limitation of this study is the incomplete availability of coagulation parameters such as D-dimer and fibrinogen. Although these biomarkers could provide valuable insight into sepsis-related coagulopathy and its association with mortality, they were not consistently available for all patients and thus could not be included in the multivariable analysis. Future studies should incorporate these and other hemostatic variables into predictive models to better elucidate the contribution of coagulation imbalance to AKI outcomes.
5. The study did not include a direct evaluation of the causes of AKI, prerenal, intrinsic or postrenal. Given the hospital-based nature of the cohort, it is plausible that many cases were related to non-kidney-specific conditions. This limits the interpretation of causality and underlines the importance of incorporating etiological characterization in future research.
6. Although environmental factors were not part of the present study design, their relevance to AKI should not be underestimated. In the rural region of Atlixco, Puebla, located within the influence area of the Alto Atoyac Basin, high environmental contamination levels may contribute to an increased prevalence of renal injury [13]. In this area, AKI may be partially driven by environmental exposure to volcanic ash from Popocatépetl and by the frequent use of agricultural pesticides associated with local economic activities. Previous studies conducted in the Atlixco, Puebla region have documented the presence of heavy metals (e.g., Pb, Cr, Cd) in volcanic ash and its leachates [75]. Although some of these elements may occur naturally in soil and aquifers, volcanic emissions can increase their environmental burden and bioavailability through contact with water, facilitating incorporation into biological cycles [76]. Pesticide residues has been documented, and these have been linked to genetic damage in local agricultural workers [77]. In Hunan Province, China—an area with high exposure to metals due to mining activity—elevated urinary copper concentrations (>20.92 μg/L) have been linked to abnormal estimated glomerular filtration rate [78]. Similarly, in Taiwan, high urinary copper levels were associated with eGFR < 60 mL/min/1.73 m^2^ [79]. In northern–central Mexico, in mining areas, chromium exposure has shown a dose-dependent relationship with increased urinary KIM-1, an early biomarker of renal damage [80]. These findings suggest that populations chronically exposed to heavy metals are at increased risk of renal impairment. Future prospective studies should therefore incorporate environmental exposure data to better delineate its contribution to AKI development in vulnerable rural populations.

## 5. Conclusions

This study identified clinical characteristics in patients with acute kidney injury (AKI), revealing alterations such as elevated inflammatory indices (NLR, MLR, and PLR), increased levels of glucose, urea, and C-reactive protein (CRP), as well as decreased lymphocyte counts, serum albumin, FiO_2_, and BCR ratio. Additionally, the hematological profile revealed a predominance of myeloid cells, characterized by neutrophilia and reduced counts of eosinophils, erythrocytes, and monocytes, consistent with systemic inflammation. Factors associated with mortality risk included COVID-19 infection, thrombocytopenia, low eosinophil levels, and polypharmacy.

Collectively, these results generate hypotheses regarding the potential role of inflammatory, metabolic, and hematological markers in the progression and mortality associated with AKI.

## Figures and Tables

**Table 1:** Overview of the classification and laboratory values of the patients with AKI.

Variables	Stage I	Stage II	Stage III	Total	
**General Characteristics**					
Number of Patients (%)	66 (62.3%)	16 (15.1%)	24 (22.6%))	106 (100%)	
18–30 years (%)	5 (4.7%)	-	-	5 (4.7%)	
31–60 years (%)	22 (20.8%)	6 (5.7%)	7 (6.6%)	35 (33.0%)	
61–90 years (%)	39 (36.8%)	10 (9.4%)	17 (16.0%)	66 (62.3%)	
Male (%)	33 (31.1%)	7 (6.6%)	15 (14.2%)	55 (51.9%)	
Female (%)	33 (31.1%)	9 (8.5%)	9 (8.5%)	51 (48.1%)	
Recovery (%)	49 (46.2%)	7 (6.6%)	6 (5.7%)	62 (58.5%)	
Death (%)	17 (16.0%)	5 (4.7%)	13 (12.3%)	35 (33.0%)	
Voluntary discharge (%)	-	4 (3.8%)	5 (4.7%)	9 (8.5%)	
**Comorbidities and polypharmacy**					
COVID-19 (%)	43 (40.6%)	5 (4.7%)	9 (8.5%)	57 (53.8%)	
Polypharmacy (%)	28 (26.4%)	9 (8.5%)	14 (13.2%)	51 (48.1%)	
HT (%)	23 (21.7%)	9 (8.5%)	15 (14.2%)	47 (44.3%)	
Diabetes (%)	26 (24.5%)	5 (4.7%)	14 (13.2%)	45 (42.5%)	
Overweight (%)	8 (7.5%)	3 (2.8%)	4 (3.8%)	15 (14.2%)	
rUTI (%)	8 (7.5%)	3 (2.8%)	3 (2.8%)	14 (13.2%)	
**Laboratory Data**					**Reference values**
Creatinine at Admission (mg/dL)	1.12 ± 0.33 **	1.79 ± 0.54 ****	3.95 ± 2.01 ****	1.86 ± 1.53	0.5–0.9
2nd Creatinine follow-up (mg/dL)	1.04 ± 0.29 **	1.87 ± 0.49 ****	3.33 ± 1.43 ****	1.63 ± 1.15	0.5–0.9
3rd Creatinine follow-up (mg/dL)	0.84 ± 0.26	1.92 ± 0.46 ****	3.01 ± 1.86 ****	1.44 ± 1.26	0.5–0.9
Total Leukocytes (10^3^/uL)	9.91 ± 6.95 *	9.88 ± 5.14	12.03 ± 5.37 ***	10.37 ± 6.40	4.8–10
Erythrocytes (10^6^/uL)	4.26 ± 0.83 ****	3.87 ± 0.88 ****	4.27 ± 0.76 ****	4.20 ± 0.83	4.59–6.50
Neutrophils (%)	83.64 ± 7.77 ****	77.93 ± 12.50 ****	84.73 ± 6.39 ****	83.02 ± 8.57	44–63.9
Lymphocytes (%)	9.77 ± 5.73 ****	15.80 ± 10.64 ****	9.77 ± 4.77 ****	10.68 ± 6.79	24–42
Monocytes (%)	4.95 ± 3.40 ****	5.00 ± 2.74 ***	4.10 ± 3.08 ****	4.76 ± 3.23	5.8–9.6
Eosinophils (%)	0.35 ± 0.72 ****	0.65 ± 1.10 ****	0.55 ± 0.85 ****	0.44 ± 0.82	1.0–4.3
Basophils (%)	1.11 ± 1.30 *	0.41 ± 0.64	0.57 ± 0.55	0.88 ± 1.13	0.0–1.3
NLR	14.04 ± 15.12 ****	8.97 ± 9.68 *	11.04 ± 5.94 ***	12.61 ± 12.95	1–3 [24,25,26,27]
MLR	0.62 ± 0.57 ****	0.44 ± 0.34	0.46 ± 0.37	0.56 ± 0.50	0.2–0.4 [25]
Hemoglobin (g/dL)	13.18 ± 2.95 ***	12.08 ± 3.06 ****	12.70 ± 2.50 ***	12.90 ± 2.87	12.8–16.7
Hematocrit (%)	36.83 ± 7.19 ****	35.44 ± 8.75 ****	36.34 ± 7.41 ****	36.51 ± 7.43	40.5–52
Platelets (10^3^/uL)	287.3 ± 132.3	243.5 ± 100.2	225.9 ± 106.7 **	266.8 ± 124.4	150–450
PLR	451.6 ± 375.8 ****	276.2 ± 287.6	234.8 ± 118.5	377.4 ± 335.8	100–200 [25]
Glucose (mg/dL)	152.7 ± 84.85 ****	140.6 ± 66.69	196.8 ± 134.0 ****	160.8 ± 97.08	85–100
Urea (mg/dL)	52.17 ± 31.32 **	53.03 ± 23.24	125.60 ± 71.36 ****	68.38 ± 51.92	16.60–49.50
BUN (mg/dL)	24.82 ± 14.35 **	30.14 ± 16.51 **	56.13 ± 35.14 ****	32.51 ± 24.37	8–23
BCR	19.08 ± 10.73	13.73 ± 6.776 *	13.24 ± 6.897 ***	16.69 ± 10.01	20
CRP (mg/L)	91.60 ± 99.37 ****	69.81 ± 69.03	161.9 ± 71.71 ****	104.6 ± 96.20	<15
Fibrinogen (mg/dL)	765.8 ± 400.6 ****	680.6 ± 461.2 *	1137 ± 273.3 ****	809.7 ± 406.0	200–400
Serum Calcium (mmol/L)	8.40 ± 1.24 ****	8.66 ± 1.11	8.40 ± 0.51 ***	8.43 ± 1.07	8.2–10.2
Serum Albumin (g/dL)	3.21 ± 0.76 ****	3.60 ± 0.85 **	2.85 ± 0.53 ****	3.15 ± 0.74	4–5
Serum Chloride (mmol/L)	100.6 ± 8.62	103.9 ± 5.07	101.1 ± 22.91	101.1 ± 13.31	98–110
pH	7.40 ± 0.14	7.37 ± 0.13	7.32 ± 0.13 **	7.37 ± 0.14	7.35–7.45
pCO_2_ (mmHg)	35.08 ± 20.82	28.13 ± 10.91	27.88 ± 12.94 *	31.66 ± 17.39	35–45
pO_2_ (mmHg)	78.46 ± 46.76	66.00 ± 13.44	76.93 ± 20.74 *	75.96 ± 36.20	>60
HCO_3_ (mmol/L)	19.89 ± 5.54 ****	16.10 ± 4.10 ****	14.32 ± 4.47 ****	17.50 ± 5.55	22–29
Oxygen Saturation (%)	82.00 ± 25.53	89.33 ± 6.63	92.41 ± 5.81	86.67 ± 18.98	>90
FiO_2_ (%)	57.10 ± 27.69 ****	24.00 ± 4.64	45.14 ± 27.25 ***	48.17 ± 27.57	21

* *p* ≤ 0.05; ** *p* ≤ 0.01; *** *p* ≤ 0.001; **** *p* ≤ 0.0001. Values are expressed as means ± standard deviation (SD). *n* = 106 patients with AKI. Percentages represent the proportion of patients in relation to the total sample. AKI: Acute kidney injury; rUTI: Recurrent urinary tract infection; HT: Hypertension; NLR: Neutrophil-to-lymphocyte ratio; MLR: Monocyte-to-lymphocyte ratio; PLR: Platelet-to-lymphocyte ratio; BUN: Blood Ureic Nitrogen; BCR: BUN-to-creatinine ratio; CRP: C-reactive protein; FiO_2_: Fraction of inspired oxygen. Minor discrepancies (up to 0.1%) may be observed due to rounding values to one decimal place.

**Table 2:** Multivariable analysis of factors associated with AKI-related mortality.

Variable	aOR	CI95-Low	CI95-High	*p*-Value	Sig
Age > 60	2.58	0.72	9.28	0.1464	
Diabetes	0.96	0.29	3.15	0.9487	
Hypertension	1.08	0.34	3.48	0.897	
Polypharmacy	3.86	1.15	12.97	0.0289	*
Stage III	8.24	1.76	38.57	0.0074	*
Low Eosinophils	22.46	2.18	231.61	0.0089	*
Low Platelets	7.06	1.71	29.18	0.0069	*
High Urea	1.88	0.53	6.7	0.3277	
COVID-19	3.68	1.1	12.29	0.0343	*

* p ≤ 0.05; aOR: Adjusted odds ratios; sig: significance.

## Data Availability

The data generated in the present study may be requested from the corresponding authors.

## Abbreviations

The following abbreviations are used in this manuscript:

AIN	Acute Interstitial Nephritis
AKI	Acute kidney injury
AKIN	Acute Kidney Injury Network
ATIN	Acute tubulointerstitial nephritis
AUC	Areas under the curve
BCR	Blood urea nitrogen-to-creatinine ratio
BUN	Blood urea nitrogen
CDK	Chronic kidney disease
CI	Confidence intervals
CRP	C-reactive protein
CRRT	Continuous renal replacement therapy
CV	Cardiovascular
Cys-C	Cystatin C
EPO	Erythropoietin
FiO_2_	Fraction of inspired oxygen
GFR	Glomerular filtration rate
HCO_3_	Bicarbonate
HGZ05	Hospital General de Zona no. 5
HT	Hypertension
ICU	Intensive care unit
IGFBP-7	Insulin-like Growth Factor-Binding Protein 7
IL-18	Interleukin 18
KDIGO	Kidney Disease Improving Global Outcomes
KIM-1	Kidney Injury Molecule-1
L-FABP	Liver-type Fatty Acid Binding Protein
MLR	Monocyte-to-lymphocyte ratio
NGAL	Neutrophil Gelatinase-Associated Lipocalin
NLR	Neutrophil-to-lymphocyte ratio
NPV	Negative Predictive Value
NSAIDs	Nonsteroidal anti-inflammatory drugs
NTUH	National Taiwan University Hospital
OR	Odd ratio
PBE	Peripheral blood eosinophilia
pCO_2_	Partial pressure of carbon dioxide
PLR	Platelet-to-lymphocyte ratio
pO_2_	Partial pressure of oxygen
PPIs	Proton pump inhibitors
PPV	Positive Predictive Value
RIFLE	Risk, Injury, Failure, Loss of Renal Function, and End-stage Renal Disease
ROC	Receiver operating characteristic
RRT	Renal replacement therapy
rUTI	Recurrent urinary tract infections
SD	Standard deviation
TIMP-2	Tissue Inhibitor of Metalloproteinase 2
WHO	World Health Organization

## Appendix A

**Table A1:** Univariable analysis of factors associated with in-hospital mortality in patients with AKI.

Comorbidity	OR	95% CI	*p*-Value
Stage I	0.2506	0.1017–0.6176	0.0031 **
Stage II	1.3100	0.3823–4.4850	0.7517
Stage III	5.5150	1.8620–16.3400	0.0025 **
18–30 years	0.1472	0.0078–2.7460	0.1558
31–60 years	0.4239	0.1598–1.1250	0.1096
61–90 years	3.2940	1.2520–8.6710	0.0158 *
Male	0.9926	0.4331–2.2750	1.0000
Female	1.0070	0.4396–2.3090	1.0000
COVID-19	1.5870	0.6797–3.7030	0.2979
Polypharmacy	2.3430	1.0000–5.4900	0.0586
HT	1.9250	0.8287–4.4720	0.1387
Diabetes	1.4280	0.6155–3.3130	0.5182
Overweight	0.7599	0.2158–2.6750	0.7645
rUTI	1.6260	0.4996–5.2900	0.5366
High Creatinine at Admission	2.0870	0.6914–6.3000	0.2109
High 2nd Control	1.8690	0.5447–6.4110	0.3949
High 3rd Control	3.4240	1.2270–9.5510	0.0253 *
Low Total Leukocytes	3.3910	0.7572–15.1800	0.1267
High Total Leukocytes	0.8025	0.3413–1.8870	0.6691
Low Erythrocytes	1.1790	0.4850–2.8680	0.8234
High Neutrophils	0.5574	0.0337–9.2030	1.0000
Lymphopenia	0.5500	0.0739–4.0880	0.6180
Low Monocytes	0.9308	0.3935–2.2020	1.0000
High Monocytes	0.2222	0.0410–1.2030	0.1241
Low Eosinophils	4.2450	0.8971–20.0900	0.0764
High Basophils	1.0420	0.3679–2.9500	1.0000
High NLR	0.5500	0.0739–4.0880	0.6180
Low MLR	0.6944	0.2227–2.1660	0.5903
High MLR	0.4211	0.1804–0.9830	0.4211
Low Hemoglobin	2.3750	1.0170–5.5450	0.0569
High Hemoglobin	1.5830	0.3906–6.4180	0.7239
Low Hematocrit	1.7290	0.6696–4.4630	0.3561
Low Platelets	4.8700	1.6310–14.5400	0.0054 **
High Platelets	0.0793	0.0044–1.4070	0.0242 *
Low PLR	2.6220	0.5507–12.4900	0.2400
High PLR	0.5018	0.1929–1.3050	0.2112
Hypoglycemia	0.5018	0.1929–1.3050	0.2112
Hyperglycemia	0.7664	0.2893–2.0300	0.6202
High Urea	3.3730	1.3560–8.3930	0.0099 **
High BUN	2.6480	1.0840–6.4670	0.0338 *
Low BCR	0.6818	0.2703–1.7200	0.4751
High BCR	1.7660	0.6867–4.5410	0.3246
High CRP	2.6250	0.4603–14.9700	0.4339
Low Fibrinogen	4.5450	0.3718–55.5800	0.2532
High Fibrinogen	0.7937	0.1574–4.0010	1.0000
Low Serum Calcium	2.1250	0.7619–5.9270	0.1889
High Serum Calcium	0.1677	0.0086–3.2420	0.2898
Low Serum Albumin	3.3540	0.6297–17.8700	0.1670
Low Serum Chloride	0.4589	0.1500–1.4040	0.2016
High Serum Chloride	4.8180	1.1050–21.0100	0.0544
Acidemia	27.5000	3.1240–242.1000	0.0002 ***
Alkalemia	0.3765	0.0980–1.4460	0.2047
Low pCO_2_	0.7619	0.1878–3.0900	0.7339
High pCO_2_	5.8820	0.6036–57.3300	0.1576
Low PO_2_	3.3170	0.9638–11.4200	0.0716
Low HCO_3_	3.5000	0.6422–19.0800	0.1596
Low Oxygen Saturation	5.0560	1.4580–17.5300	0.0173 *
High FiO_2_	2.3330	0.4990–10.9100	0.4604

ORs and 95% Cis for demographic and clinical variables including age (18–30, 31–60, and 61–90 years), AKI stage (I, II, III), sex, and comorbidities (hypertension (HT), polypharmacy, COVID-19, recurrent urinary tract infections (rUTI), diabetes, and overweight). * *p* ≤ 0.05; ** *p* ≤ 0.01; *** *p* ≤ 0.001.

**Table A2:** Performance metrics of the multivariable logistic regression model.

Metric	Value	Interpretation
AUC-ROC	0.873	Excellent discrimination
Bootstrap AUC (95% CI)	0.872 (0.798–0.936)	Robust performance under resampling
Cross-validation AUC (5-fold)	0.796 ± 0.145	Acceptable model stability
Brier score	0.139	Good calibration (low prediction error)
Optimal threshold (Youden index)	0.403	Optimal cutoff for binary classification
Sensitivity	0.857	Correctly identifies 85.7% of deceased patients
Specificity	0.823	Correctly identifies 82.3% of survivors
Positive predictive value (PPV)	0.732	Probability of death among high-risk predictions
Negative predictive value (NPV)	0.911	Probability of survival among low-risk predictions
